# RANBP1, a member of the nuclear-cytoplasmic trafficking-regulator complex, is the terminal-striking point of the SGK1-dependent Th17^+^ pathological differentiation

**DOI:** 10.3389/fimmu.2023.1213805

**Published:** 2023-06-27

**Authors:** Carolina Brescia, Vincenzo Dattilo, Lucia D’Antona, Emanuela Chiarella, Rossana Tallerico, Salvatore Audia, Valentina Rocca, Rodolfo Iuliano, Francesco Trapasso, Nicola Perrotti, Rosario Amato

**Affiliations:** ^1^ Department of Health Science, Medical School, University “Magna Graecia” of Catanzaro, Catanzaro, Italy; ^2^ Immuno-Genetics Lab, Department of Health Science, Medical School, University “Magna Graecia” of Catanzaro, Catanzaro, Italy; ^3^ Department of Experimental and Clinical Medicine, Medical School, University “Magna Graecia” of Catanzaro, Catanzaro, Italy; ^4^ Medical Genetics Unit, University Hospital, Medical School, University “Magna Graecia” of Catanzaro, Catanzaro, Italy; ^5^ Microbiology and Virology Unit, “Pugliese-Ciaccio” Hospital, Catanzaro, Italy

**Keywords:** Th17^+^, nuclear transport, SGK1, RANBP1, FOXO1

## Abstract

The Th17^+^ arrangement is critical for orchestrating both innate and acquired immune responses. In this context, the serum and glucocorticoid regulated kinase 1 (SGK1) exerts a key role in the governance of IL-23R-dependent Th17^+^ maturation, through the phosphorylation-dependent control of FOXO1 localization. Our previous work has shown that some of the SGK1-key functions are dependent on RAN-binding protein 1 (RANBP1), a terminal gene in the nuclear transport regulation. Here, we show that RANBP1, similarly to SGK1, is modulated during Th17^+^ differentiation and that RANBP1 fluctuations mediate the SGK1-dependent effects on Th17^+^ maturation. RANBP1, as the final effector of the SGK1 pathway, affects FOXO1 transport from the nucleus to the cytoplasm, thus enabling RORγt activation. In this light, RANBP1 represents the missing piece, in an essential and rate-limiting manner, underlying the Th17^+^ immune asset.

## 1 Introduction

T-helper-17 (Th17^+^) population is a subset of T-helper (CD4^+^) cells with pro-inflammatory features, defined by the expression, among other cytokines, of IL-17A (1). The expansion and maintenance of differentiation characteristics of the CD4^+^Th17^+^ population depend on differentiation factors (transforming growth factor β, TGF-β), growth factors (IL-23/IL-23R), and several transcription factors (RORγt and STAT3) (2, 3). Th17^+^ lymphocytes play a key role in autoimmune diseases, tumor drug resistance, tumor microenvironment definition, and HIV infection/progression (4–6). Recent data show that a high salt concentration is able to trigger Th17^+^ switch, defined by increased IL-17A production, low IL-10 level (7), and subsequent Treg down-regulation, thus favoring a microenvironmental subthreshold inflammation (8). Salt plays a crucial but still controversial role in immunoregulatory processes (9). Although chronic NaCl stimulations result in a significant Th17^+^ switch, the immune cell phenotype remains a topic of debate (10). It remains unclear whether Th17^+^ cells induced by NaCl stimulation play a pro-inflammatory or negative regulatory role, or both, depending on their specific maturation, given the intricate complexity of Th17^+^ plasticity (11). The IL-23/IL-23R interaction plays a key role in the establishment of this phenotype (12, 13). From a molecular point of view, hypertonicity of the medium, as obtained by high salt concentration, leads to p38/MAPK phosphorylation and to the activation of downstream-targets, particularly NFAT5, which, in turn, induces SGK1, a kinase involved in neoplastic (14, 15), metabolic, and immune processes (16). SGK1 stabilizes the expression of IL-23R, thereby stimulating the retinoid-related orphan receptor-γ (RORγt) activity, a final regulator of Th17^+^ differentiation (17). Similarly, in murine models, it was demonstrated that TGF-β, combined with IL-6, primed differentiation of naїve CD4^+^ T (Th0) cells to Th17^+^ cells (18, 19). These cytokines induce the transcription factor RORγt, which, cooperating with RORα and other transcription factors, promotes the expression of IL-17A and IL-23R on maturating Th17^+^ cells (20). Our previous data, obtained *in vivo models*, demonstrated that SGK1 contributed to Th17^+^ transition in an experimental system of ulcerative colitis and chronic venous disease, with obvious pathological effects on disease targets (21, 22). SGK1 appears to play a critical role in the modulation of Treg/Th17 phenotype, through phosphorylation-dependent control of the nuclear-cytoplasmic FOXO1 localization (23, 24). SGK1, unique among AGC family kinases, phosphorylates SP1 and activates the expression of RANBP1, with consequences in terms of mitotic stability and nuclear transport (25). RANBP1 is a main regulator of the RAN complex, implicated in the control of nuclear transport and mitotic integrity (26). In the present work we wondered whether RANBP1 had a role in Th17^+^ maturation by mediating the SGK1 dependent FOXO1 nucleus-cytoplasmic translocation and found, in primary human-derived samples, that RANBP1 is indeed the key element in SGK1-dependent FOXO1 nuclear translocation, thus affecting and controlling Th17^+^ differentiation.

## 2 Materials and methods

All reagents and resources with sources and univocal identifiers, used in the whole experimental process, are summarized in **Supplementary Table 1**.

### 2.1 *In vitro* T cell differentiation

CD4^+^ T lymphocytes were isolated from PBMCs (buffy coat of 50 ml whole blood) by using a magnetic cell sorting CD4^+^ T cell isolation kit (Miltenyi Biotec), according to the manufacturer’s instructions. Once isolated, cells were counted and seeded in six-well plates at a density of 1 x 10^6^ cells/mL. Cells were cultured in RPMI 1640 (NaCl 0.08 mEq./mL) complete medium with 5% FBS and 1% streptomycin/penicillin at 37°C in a humidified atmosphere with 5% CO_2_ and 95% air. CD4^+^ T cells were then induced to differentiate in Th17^+^ cells by the addition in the culture medium of 25 ng/mL IL-6 (Sigma-Aldrich), 5 ng/mL TGF-β1 (Sigma-Aldrich), and Dynabeads Human T-Activator CD3/CD28 (Life Technologies), in the presence/absence of 40 mM NaCl (0.1 mEq./mL) and for a time of differentiation of 5 or 20 days. CD4^+^ T naïve (Th0) were maintained in culture by the addition in the culture medium of 10 ng/mL IL-2 (Sigma-Aldrich).

### 2.2 Plasmids and lentiviral production

A plasmid containing the wild-type SGK1 sequence (pHIV-EGFP-SGK1) was obtained as previously described (27). For RANBP1, the wild-type coding sequence within pEGFP-N1-RANBP1, kindly provided by Dr. Lavia P. (28), was sub-cloned into the same expression vector used for SGK1 over-expression (pHIV-EGFP-RANBP1) by using *XhoI* and *BamHI* restriction sites. pHIV-EGFP-SGK1, pHIV-EGFP-RANBP1, or pHIV-EGFP (negative control) were used to generate lentiviral particles in HEK293T packaging cells, as previously reported (29). The active transduction of CD4^+^ T lymphocytes was performed by adding 6 μg/mL polybrene (Sigma-Aldrich), followed by Spin-inoculation at 1,900 rpm, for 40 min, at 32°C. RANBP1 silencing was obtained through lentiviral particles (sc-41848-V Santa Cruz) in the presence of 6 μg/mL polybrene (Sigma-Aldrich) according to manufacturer’s instructions; ShScr (Sigma-Aldrich), employed to generate control lentiviral particles, was utilized as previously reported (29).

### 2.3 Flow cytometry

For cytofluorimetric analysis, CD4^+^ T naïve (Th0) and Th17^+^ lymphocytes were stimulated for 4 hours with 50 ng/mL phorbol 12-myristate 13-acetate (PMA) (Sigma-Aldrich) and 1 μM ionomycin (Sigma-Aldrich) in the presence of 10 μg/mL of the protein transport inhibitor Brefeldin A (BFA) (Sigma-Aldrich). Cells were fixed and permeabilized with 300 μL with BD Cytofix/Cytoperm solution (BD Biosciences) and then stained with PE anti-CD4 (Biolegend), FITC anti-CD4 (MiltenyiBiotec), Cy5.5 anti-RANBP1 (Bioss Antibodies), PE-A anti-IL17A (MiltenyiBiotec), and APC anti-SGK1 (Santa Cruz) antibodies for 45 min at 4°C. Subsequently the cells were washed twice with PBS and acquired by FACSCanto II. The data were analyzed by FlowJo software 8.8.6 (BD Biosciences).

### 2.4 RNA extraction and quantitative real-time PCR

Total RNA was extracted using RNeasy Mini Kit (Qiagen, Valencia, CA, USA) according to the manufacturer’s instructions. RNA was then quantified using NanoDrop 2000/2000c spectrophotometer (ThermoFisher, Waltham, MA, USA) and 1 μg was reverse transcribed using the High-Capacity RNA-to-cDNA kit (Applied Biosystems, Foster City, CA, USA) according to the manufacturer’s instructions. One microliter of cDNA was amplified via real-time PCR using GoTaq qPCR Master Mix (Promega) and 10 pmol of primers specific for SGK1, RANBP1, IL-17A, IL-23R, RORγt, IL-10, FOXP3, and HPRT1, the latter used as a housekeeping gene. The primer list can be found in the key resources table. Real-time PCR assays were performed in triplicate in a total volume of 20 μL using a CFX96 Touch Real-Time Detection System under the following conditions: initial denaturation for 3 minutes at 95°C, followed by 40 cycles of 10 seconds at 95°C and 1 minute at 57°C. The specificity of the PCR products was determined via melting curve analysis. The fold changes were calculated using the 2^-ΔΔCt^ threshold cycle method.

### 2.5 Immunoblot analysis

CD4^+^ T naïve (Th0) and Th17^+^ lymphocytes were collected for protein extraction using lysis buffer (50 mM Tris-HCl, pH 7.4, 0.15 M NaCl, 0. 5% IGEPAL CA-630, 25 mM NaF, 1 mM DTT, 1 mM Na_3_VO_4_, 2mM PMSF, and 8 nM Aprotinin). Whole-cell lysates were separated via SDS page by using 4-12% NuPAGE precast gels (ThermoFisher) and transferred to a 0.2 μm PVDF membrane via Trans-Blot Turbo Transfer Pack (Bio-Rad). Membrane blocking was performed with 1X TPBS, 0.1% Tween-20 with 5% w/v skimmed milk powder for 1 h at room temperature and incubated overnight with antibodies, namely RORγt (1:1000 Santa Cruz Biotechnology), RANBP1 (1:1000 Santa Cruz), IL-23R (1:1000 ThermoFisher), SGK1 (1:1000 Millipore), p-SGK1 (1:1000 Cell Signaling Technology), FOXO1 (1:1000 ThermoFisher), p-FOXO1 (1:1000 ThermoFisher), FOXP3 (1:1000 Cell Signaling Technology), and GAPDH (1:1000 Santa Cruz Biotechnology), the latter used as loading control. All primary antibodies were detected with appropriate HRP-conjugated secondary antibodies. Detection was performed using ECL reagent (Cytiva) and chemiluminescence signals were recorded using Uvitec Cambridge. For nuclear-cytoplasm protein extraction, cellular pellets were first put in contact with a lysis buffer for cytoplasmic protein extraction (HEPES 20mM, Igepal CA-630 20%, DTT 0.5mM, MgCl_2_ 1. 5mM, KCl 10mM, NaF 1mM, Na_3_VO_4_ 1mM, Aprotinin 8nM, and PMSF 2mM), kept for 10 minutes on ice, and centrifuged at 10,000 rpm for 1 minute. After centrifugation, the supernatant containing the cytoplasmic proteins was collected. Then, the cellular pellets were placed in contact with a lysis buffer for nuclear protein extraction (HEPES 20mM, NaCl 420mM, 25% glycerol, DTT 0.5mM, MgCl_2_ 1.5mM, EDTA 2mM, NaF 1mM, Na_3_VO_4_ 1mM, Aprotinin 8nM, and PMSF 2mM), kept on ice for 20 minutes, and centrifuged at 13,000 rpm for 15 minutes. After centrifugation, the supernatant containing the nuclear proteins was collected. Antibodies against α-Tubulin (1:1000 Santa Cruz Biotechnology) and Nucleolin (1:1000 Cell Signaling Technology) were used as cytoplasmic and nuclear loading controls, respectively. Detection was performed using ECL reagent (Cytiva) and chemiluminescence signals were recorded using Uvitec Cambridge.

### 2.6 Immunofluorescence

After activation, CD4^+^ T naïve (Th0) and Th17^+^ cells were collected and placed on a sample chamber and centrifuged via Shandon Cytospin 4 cytocentrifuge (ThermoFisher) at 400 rpm for 4 minutes. After centrifugation, cells were fixed with ice-cold 4% PFA, permeabilized in 0.5% Triton, and washed with phosphate-buffered saline (PBS) (pH 7.4). Staining was performed using blocking solution (Triton 0.1% and BSA 1%) with the following primary antibodies: FOXO1 rabbit polyclonal antibody (1:250 ThermoFisher) and RANBP1 goat polyclonal antibody (1:250 Santa Cruz Biotechnology). The following secondary antibodies were then used: Alexa Fluor 594 AffiniPure Donkey Anti-Rabbit IgG (H+L) and Alexa Fluor 594 AffiniPure F(ab’)_2_ Fragment Donkey Anti-Goat IgG (H+L) (1:500 Jackson Immunoresearch). The nuclei were highlighted with 4’,6-diamidine-2-phenylindole (DAPI 0.05% μg/mL, Sigma-Aldrich). Fluorescence and images were acquired with a Leica TCS SP8 confocal microscope and processed with Leica confocal software. The digital zoom is indicated by the scale bar.

### 2.7 Statistical analysis

All tests were performed at least in triplicate, and all experiments were performed at least three times, unless otherwise specified (biological triplicate). Each biological triplicate results from the mean of three technical triplicates. The results are expressed as the mean ± standard deviation (SD). Differences between two groups were analyzed using unpaired two-tailed Student’s t-tests. Multiple-group comparisons were performed with one-way analysis of variance (ANOVA), followed by Bonferroni’s *post hoc* test. Immunofluorescence group analyses for FOXO1 and RANBP1 localization were performed by Chi-square at two-sided analysis. The analysis was conducted using GraphPad Prism software (San Diego, CA, USA), and differences were considered significant at *p ≤ 0.05, **p ≤ 0.01, and ***p ≤ 0.001.

## 3 Results

### 3.1 IL-17 expression in Th17^+^ lymphocytes differentiation

**Figure 1:**
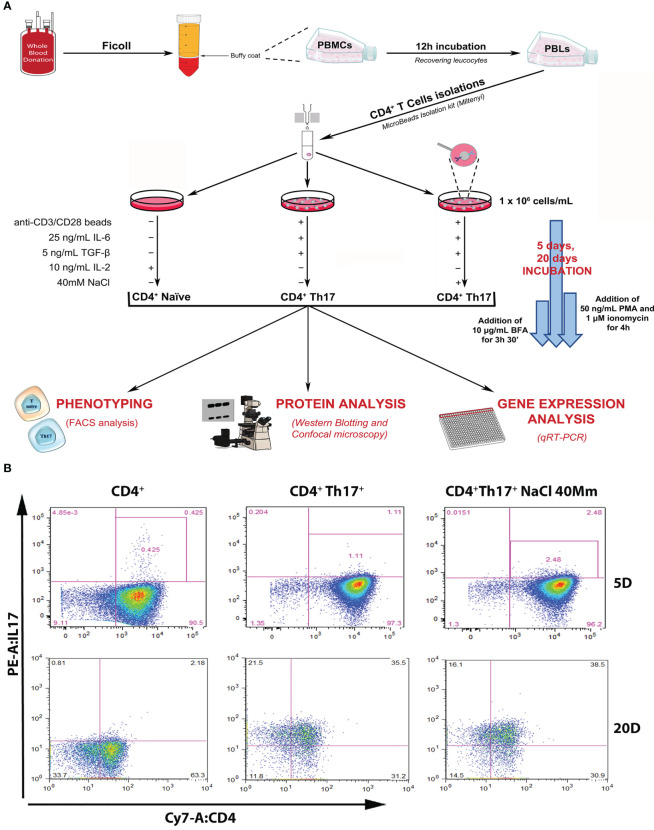
Experimental design and setting. **(A)** Schematic representation of the experimental design, leading to the isolation of CD4^+^ naïve (Th0) lymphocytes from the healthy donor’s buffy coat, subsequent Th17^+^ differentiation procedures, and scientific data analysis. **(B)** Flow cytometric analysis of IL-17A/CD4^+^ expression in control CD4^+^ naïve (Th0) cells, in basal and under 40mM NaCl stimulation CD4^+^Th17^+^ cells, at 5 (upper panel) and 20 (bottom panel) days of differentiation. Data are representative of three independent experimental sets (n=3) (for statistical analysis see **Figure 2C** graph-left columns and **Supplementary Figure 1A**).

**Figure 2:**
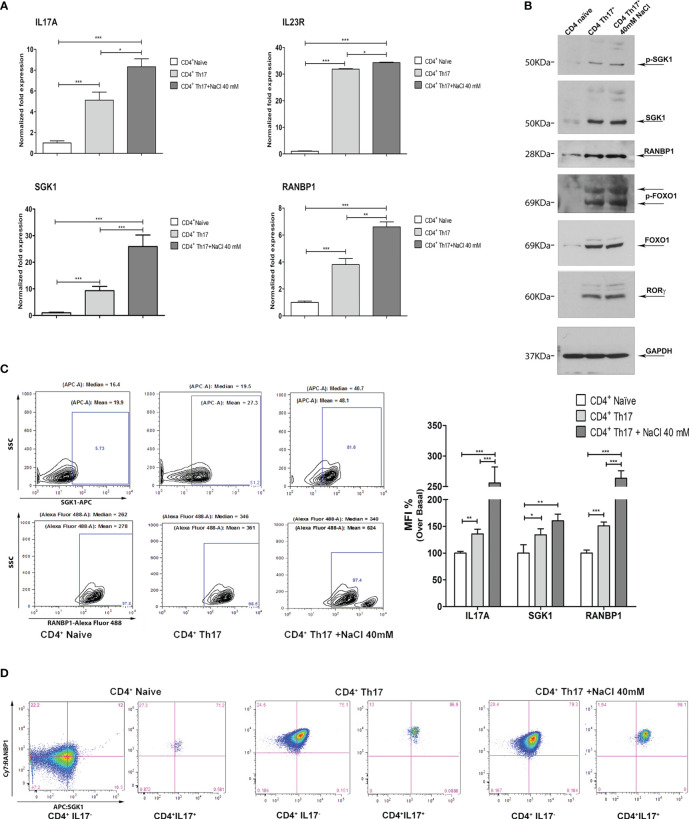
Coordinated modulation of RANBP1/SGK1 during the Th17^+^ differentiation process. **(A)** qPCR analysis showing the expression of RANBP1, SGK1, and associated Th17^+^ markers (IL-23R; IL-17A) in CD4^+^ naïve (Th0) control cells, in basal and under 40mM NaCl stimulation CD4^+^Th17^+^ cells, at 5 days of differentiation (n=5). **(B)** Immunoblot analysis of RANBP1 and phospho-SGK1/SGK1 and associated Th17^+^ markers (p-FOXO1/FOXO1 and RORγt), in CD4^+^ naïve (Th0) control cells, in basal and under 40mM NaCl stimulation CD4^+^Th17^+^ cells, at 5 days of differentiation (n=3). **(C)** Flow cytometric analysis showing RANBP1 and SGK1 expression in CD4^+^ naïve (Th0) control cells, in basal and under 40mM NaCl stimulation CD4^+^Th17^+^ cells, at 5 days of differentiation (n=3) and IL-17A, SGK1, and RANBP1 statistical analysis, referred to the top panel of **Figure 1B** (IL-17A expression at 5 days) and the left panel of **C** (RANBP1, SGK1 expression, at 5 days) (n=3). **(D)** Flow cytometric analysis of RANBP1 and SGK1 coordinated co-expression in CD4^+^ naïve (Th0) control cells, in basal and under 40mM NaCl stimulation CD4^+^Th17^+^ cells, at 5 days of differentiation (n=3). Data are representative of at least three independent experiments (unless otherwise specified) and are shown as mean ± SD. *p <0.05, **p < 0.01, and ***p < 0.001 determined by one-way ANOVA followed by Bonferroni’s *post hoc* test.

A Th17^+^ lymphocyte differentiation experiment was designed starting from primary cultures of human Th17^+^ obtained from 40 hematic buffy coats by healthy donors. All subjects gave their informed consent for inclusion and the study was approved by the local Ethics Committee. Complete anonymity was assured by coding samples. CD4^+^Th17^+^ isolation and differentiation were performed, as shown in **Figure 1A** and described in Methods Section. Following differentiation, IL-17A expression on the isolated CD4^+^ population on days 5 and 20 (start and end of the differentiation process, respectively) was verified by FACS analysis. The increase in IL-17A expression on days 5 and 20 was statistically significant in CD4 ^+^ Th17^+^ lymphocytes, both in the absence and in presence of NaCl, compared with CD4^+^ naïve cells, as expected based on the published literature (16, 17) (**Figures 1B**, **2C** graph-first columns group, **Supplementary Figure 1A**). Interestingly, the dependent NaCl contribution on IL-17A expression was evident only on day 5, at the early stage of differentiation. In later stages of differentiation, on day 20, with a presumably maximal IL-17A expression, the addition of NaCl did not result in further expression of IL-17A. We therefore focused our analysis on the early stages of Th17^+^ differentiation. Cells were kept in culture for 20 days anyway, just to confirm the maintenance of the observed phenotype, even in the late stages of the Th17^+^ maturation and expansion process.

### 3.2 RANBP1 and related pathway in Th17^+^ genic signature

In order to dissect the molecular pathway of Th17^+^ differentiation, the expression of RANBP1, SGK1, and other Th17^+^-specific markers were evaluated at the transcript and protein level. At the transcript level, on day 5 of Th17^+^ differentiation, a significant increase in the expression of IL-17A, IL-23R, SGK1, and RANBP1 has been documented. The addition of NaCl (40mM) further increased the expression of IL-17A, IL-23R, SGK1, and RANBP1 on day 5, as expected based on the published literature, at least for SGK1 (8, 17) (**Figure 2A**). At the protein level, the expression of phospho-SGK1, SGK1, RANBP1, phospho-FOXO1, FOXO1, and RORγt was assessed by western blotting. GAPDH was used to normalize protein expression data (**Figure 2B**; **Supplementary Table 2**). Similarly to what was observed at the transcript level, a substantial increase in protein expression of phospho-SGK1 (1.64 ± 0.05 A.U.), SGK1 (1.90 ± 0.01 A.U.), RANBP1 (1.96 ± 0.11 A.U.), phospho-FOXO1 (1.93 ± 0.31 A.U.), FOXO1 (1.90 ± 0.36 A.U.), and RORγt (1.91 ± 0.12 A.U.) was observed in Th17^+^ compared with naïve cells, on day 5 of differentiation. Again, the addition of NaCl (40 mM) slightly increased protein expression (p-SGK1 1.94 ± 0.13; SGK1 1.94 ± 0.03; RANBP1 2.13 ± 0.1; p-FOXO1 1.99 ± 0.2; FOXO1 1.92 ± 0.014; RORγt 1.94 ± 0,12 A.U.). Interestingly, the increase in RANBP1 protein expression parallels the expression of the main markers, involved in Th17^+^ differentiation (p-FOXO1, FOXO1, RORγt, and SGK1). From a technical point of view, we evaluated mRNA expression only for those markers whose changes at the transcriptional level are critical in the differentiation process, where, in contrast, FOXO1 and phospho-FOXO1 levels are predominantly susceptible to post-translational and maturational changes during the differentiation process. As a core concept, we can state that, during the induced CD4^+^ to Th17^+^ maturation process, similarly to what was described for mRNA changes, a strong protein increase has been recorded, not only for the main proteins involved in Th17^+^ differentiation (p-FOXO1, FOXO1, RORγt, and more recently SGK1), but also for RANBP1. These appealing results were confirmed in intact cells using an intracellular FACS approach (**Figure 2C** left). Again, a marked and significant increase in SGK1 and RANBP1 expression was confirmed in CD4^+^Th17^+^ compared with CD4^+^ naïve (Th0) control cells (**Figure 2C** middle plot Vs left plot and MFI quantification right graph). The addition of NaCl (40mM) further significantly increased the expression of SGK1 and RANBP1 compared with CD4^+^ naïve (Th0) and CD4^+^Th17^+^lymphocytes (**Figure 2C** right plot Vs left plot and MFI quantification right graph). Co-expression between SGK1 and RANBP1 in cells gated for IL-17A was analyzed in comparison with global unstained reference populations. A gradual and progressive increase in cells expressing the two proteins was detected proceeding from CD4^+^ naïve (Th0) cells to CD4^+^Th17^+^ and CD4^+^Th17^+^ + 40mM NaCl, thus suggesting a coordinated expression increase of SGK1 and RANBP1 during Th17^+^ differentiation and upon NaCl stimulation (**Figure 2D**; **Supplementary Figure 1B** for MFI quantification). Again, in Th17^+^ and Th17^+^ cells treated with 40mM NaCl, it was possible to document an important increase in RANBP1 expression, compared with control CD4^+^ cells on day 5 of differentiation (**Supplementary Figure 1B**, right column Vs middle column for MFI quantification). Interestingly, the increase in the percentage of cells expressing RANBP1 was stable during the differentiation process, since it was detected on day 20 (**Supplementary Figure 1C** and MFI quantification right graph) in CD4^+^Th17^+^ compared to CD4^+^ (Th0) control cells, thus highlighting a constitutive and not occasional role of RANBP1 during the differentiation process. As previously observed, at 20 days, despite an exponential increase of RANBP1 expression, there was no additive effect for NaCl stimulation. This apparent discrepancy can be explained if we consider a maximal RANBP1 expression in these experimental conditions that cannot be further enhanced by NaCl stimulation. Moreover, RANBP1 and SGK1 protein expression remained high especially when analyzed in IL-17^+^ population (double positive population), compared with CD4^+^Th17^+^ cells, on day 20 of differentiation (**Supplementary Figure 1D** and MFI quantification right graph). Thus, it can be concluded that for both the early and more advanced stages of the differentiation process, the expression of IL-17A, a marker of Th17^+^ differentiation, is paralleled by a concomitant expression of SGK1 and RANBP1. The importance of SGK1 expression in Th17^+^ differentiation is consistent with the published literature (16, 17), whereas the expression of RANBP1 in these experimental conditions represents a novelty of the present paper.

### 3.3 RANBP1 fluctuations are critical in SGK1-dependent signal transduction

**Figure 3:**
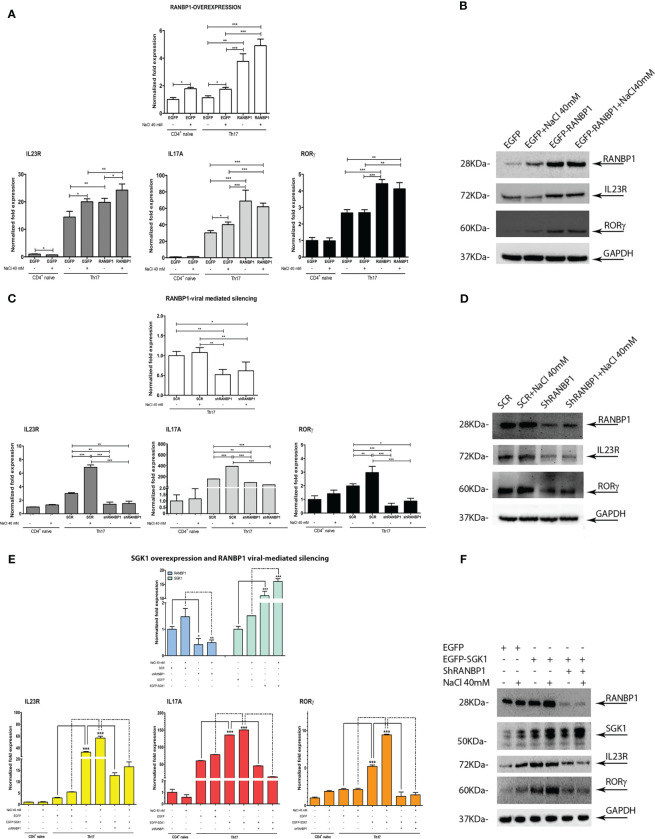
RANBP1 fluctuations affect Th17^+^ differentiation markers expression and interfere with SGK1-dependent signal transduction. **(A, B)** qPCR and immunoblot analysis to assess the expression of RANBP1 (under RANBP1 lentiviral over-expression) and associated Th17^+^ markers (IL-23R; IL-17A; RORγt for qPCR and IL-23R and RORγt for WB over GAPDH normalization) in CD4^+^ naïve (Th0) control cells, in basal and under 40mM NaCl stimulation CD4^+^Th17^+^ cells, at 5 days of differentiation (n=5 qPCR; n=3 WB). **(C, D)** qPCR and immunoblot analysis to assess the expression of RANBP1 (under RANBP1 lentiviral mediated Silencing) and associated Th17^+^ markers (IL-23R; IL-17A; RORγt for qPCR and IL-23R and RORγt for WB over GAPDH normalization) in CD4^+^ naïve (Th0) control cells, in basal and under 40mM NaCl stimulation CD4^+^Th17^+^ cells, at 5 days of differentiation (n=5 qPCR; n=3 WB). **(E, F)** qPCR and immunoblot analysis to assess the expression of RANBP1 (under SGK1 lentiviral over-expression, +/- concomitant RANBP1 lentiviral mediated silencing) and associated Th17^+^ markers (IL-23R; IL-17A; RORγt for qPCR and IL-23R and RORγt for WB over GAPDH normalization) in CD4^+^ naïve (Th0) control cells, in basal and under 40mM NaCl stimulation CD4^+^Th17^+^ cells, at 5 days of differentiation (n=5 qPCR; n=3 WB). Data are representative of at least three independent experiments (unless otherwise specified) and are shown as mean ± SD. *p <0.05, **p < 0.01, and ***p < 0.001 determined by one-way ANOVA followed by Bonferroni’s *post hoc* test.

A multiple lentiviral approach has been used to define the relative role of SGK1 and RANBP1 expression in Th17^+^ differentiation. Primary CD4^+^ naïve lymphocytes (Th0) were transduced with a lentiviral vector encoding for RANBP1 in order to obtain an enforced expression of RANBP1 during Th17^+^ differentiation (**Figure 3A** upper panel). RANBP1 over-expression was associated with increased expression of IL-23R, IL-17A, and RORγt (**Figure 3A** lower panels) compared with cells transduced with the control vector (EGFP transduced cells). NaCl addition (40 mM) resulted in enhanced expression of IL-23R and IL-17A in EGFP transduced cells and a NaCl additive effect was demonstrated only for IL-23R in RANBP1 transduced cells compared with basal RANBP1-Th17^+^ cells. The results were confirmed at the protein level by western blot: the over-expression of RANBP1 was, in fact, associated with enhanced protein expression over orthologous control cells of IL-23R (1.97 ± 0.07 A.U.) and RORγt (1.57 ± 0.01 A.U), the latter representing the final and determining element for the complete differentiative process (**Figure 3B**; **Supplementary Table 2**). In another set of experiments, primary CD4^+^ cells were infected with a lentivirus, carrying the silencing sequence of RANBP1, before initiating the differentiation process. At the transcriptional level, RANBP1 silencing abrogated about 50% of RANBP1 expression (**Figure 3C** upper panel). In terms of Th17^+^ differentiation markers, RANBP1 silencing was associated with a significant reduction in the expression of IL-23R, IL-17A, and RORγt that was not significantly affected by NaCl addition (**Figure 3C** lower panels). Again, the reduced expression, originally detected at the transcriptional level, was confirmed by western blot at the protein level for RANBP1, IL-23R (0.4 ± 0.032 A.U.), and RORγt (0.31 ± 0.011 A.U.) (**Figure 3D**; **Supplementary Table 2**). The reduced expression of IL-17A expression, an essential marker of Th17^+^ differentiation, was further confirmed by FACS analysis of RANBP1-silenced Th17^+^ lymphocytes, both at early (5 days, **Supplementary Figure 2A**) and late (20 days, **Supplementary Figure 2B**) stages. Finally, CD4^+^ were co-infected with two different vectors, used alone or in combination, inducing either SGK1 over-expression or RANBP1 silencing. SGK1 over-expression induced an enhanced expression of IL-23R, IL-17A, and RORγt, detected at the transcriptional and protein level (IL-23R basal 1.6 ± 0.2 and +NaCl 1.2 ± 0.1; RORγt basal 1.83 ± 0.12 and +NaCl 2.2 ± 0.11 A.U.), as expected. When SGK1 over-expression occurred together with RANBP1 silencing, a complete reversal of the phenotype was observed (at protein level: IL-23R basal 1.18 ± 0,2, +NaCl 0.8 ± 0.2; RORγt basal 1.2 ± 0.15, +NaCl 0.7 ± 0.31 A.U), with expression values comparable to those of Th17^+^ control cells, and a complete inhibition of the SGK1-dependent effect (**Figures 3E, F**; **Supplementary Table 2**). Taken together, our data strongly indicate that RANBP1 expression is essential and rate-limiting in the transduction of SGK1-dependent Th17^+^ differentiation signals. Recently, a difference between physiological and pathological Th17^+^ differentiation has been proposed, with opposite functional meanings and clinical implications, based on a different IL-17/IL-10 ratio (7). Briefly, a physiological Th17^+^ differentiation is characterized by a parallel increase of both IL-17 and IL-10, whereas a prevalent increase of IL-17 suggests a pathological differentiation. In order to better define the Th17^+^ differentiation pathway activated by RANBP1 expression, IL-10 was measured in Th17^+^ cells transduced with lentiviral vectors inducing RANBP1 over-expression or silencing. RANBP1 over-expression led to a marked reduction in IL-10 mRNA levels, consistent with what is presumed to be a pathological Th17^+^ differentiation, on the other hand RANBP1 silencing induced a significant over-basal increase of IL-10 transcript well-matched with a physiological Th17^+^ differentiation (**Supplementary Figure 2C**). Similar results on IL-10 expression were obtained when Th17^+^ cells were transduced with SGK1 over-expressing vectors or RANBP1 silencing vectors alone or in combination. SGK1 over-expression induced a significant decrease in IL-10 expression levels, similar to what was observed in RANBP1 over-expressing cells. However, when SGK1 over-expression was associated with RANBP1 silencing, normal IL-10 expression was restored (**Supplementary Figure 2D**). In conclusion, SGK1-RANBP1 signaling is implicated in Th17^+^ differentiation with high IL-17 and low IL-10 values, as with a pathological Th17^+^ maturation. As expected from the literature (10), stimulation with 40mM NaCl has an inducing effect on FOXP3 transcriptional levels in Th17^+^ lymphocytes (**Supplementary Figure 2E**). Interestingly, this effect is abrogated by over-expression of SGK1 or RANBP1. Of note, concurrent RANBP1 silencing and SGK1 overexpression lead to a significant increase in FOXP3 transcriptional levels at baseline, without a NaCl-dependent additive effect. Similarly, the protein analysis by Western blot (**Supplementary Figure 2F**) reveals that downregulation of RANBP1 promotes protein expression of FOXP3. However, upregulation of RANBP1 doesn’t seem to have any significant impact. This suggests that the regulation of FOXP3 expression in Th17^+^ lymphocytes involves RANBP1, which may be necessary, but not a rate-limiting factor, potentially through FOXO1 nuclear/cytoplasmic regulation.

### 3.4 RANBP1 genetic modulation alters the FOXO1 nuclear-cytoplasmic trafficking during Th17^+^ differentiation

**Figure 4:**
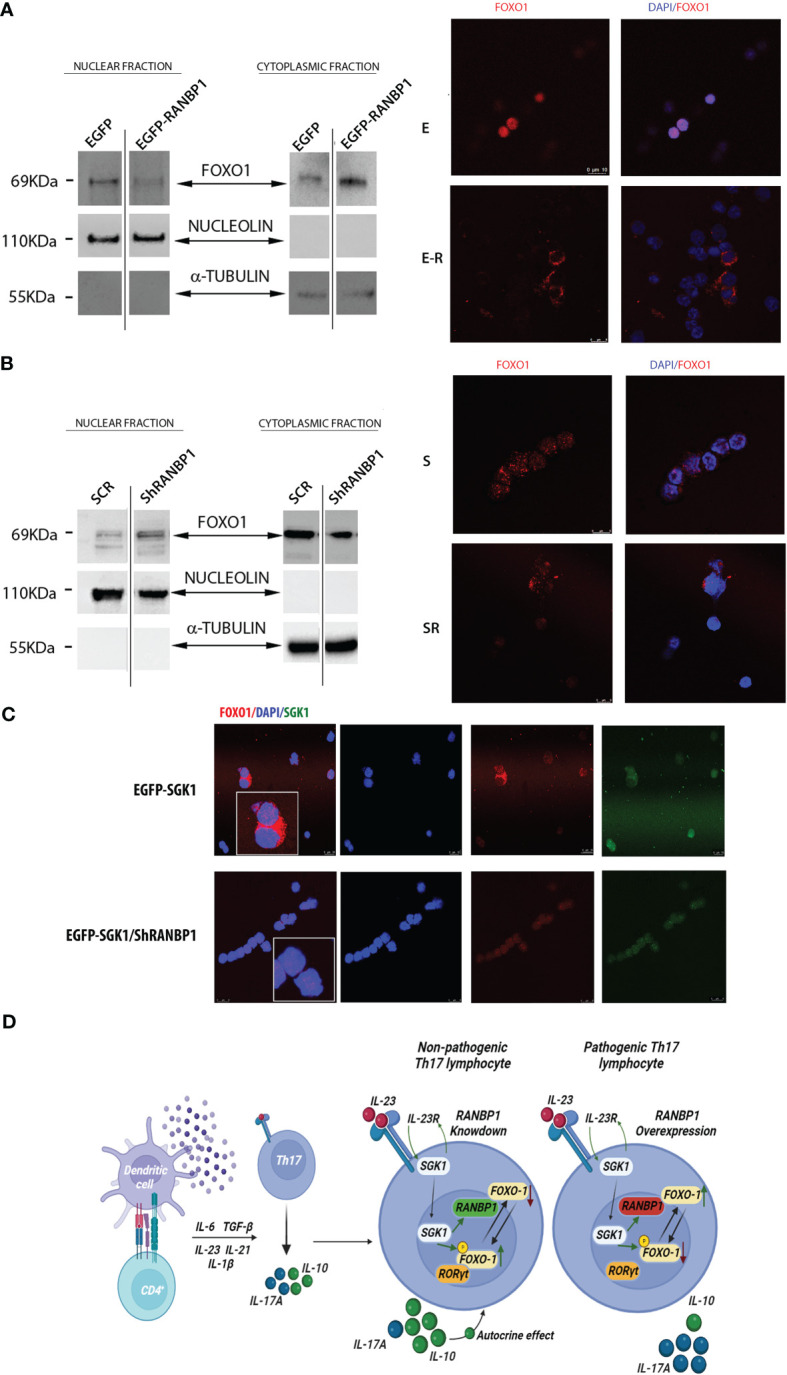
RANBP1 regulates FOXO1 nuclear exclusion during Th17^+^ differentiation, working as an SGK1 down-stream effector. **(A)** Immunoblot analysis of FOXO1 expression in the separated cytoplasmic and nuclear fractions (cropped gel, SF3 for uncropped version), under RANBP1 lentiviral over-expression in CD4^+^ naïve (Th0) control cells and in basal CD4^+^Th17^+^ cells, at 5 days of differentiation. α-Tubulin and Nucleolin were used as cytoplasmic and nuclear loading controls, respectively. Under the same experimental conditions, the localization of FOXO1 was observed by immunofluorescence (Red Ch FOXO1, Blue Ch=DAPI). (n=2 for WB and n=3 for IF). **(B)** Immunoblot analysis of FOXO1 expression in the separated cytoplasmic and nuclear fractions (cropped gel, SF3 for uncropped version), under RANBP1 lentiviral silencing in CD4^+^ naïve (Th0) control cells and in basal CD4^+^Th17^+^ cells, at 5 days of differentiation. α-Tubulin and Nucleolin were used as cytoplasmic and nuclear loading controls, respectively. Under the same experimental conditions, the localization of FOXO1 was observed by immunofluorescence (Red Ch FOXO1, Blue Ch=DAPI). (n=2 for WB and n=3 for IF). **(C)** Immunofluorescence analysis of FOXO1 under SGK1 lentiviral over-expression, +/- concomitant RANBP1 lentiviral mediated silencing, in CD4^+^ naïve (Th0) control cells and in basal CD4^+^Th17^+^ cells, at 5 days of differentiation. (Red Ch=FOXO1, Blue Ch=DAPI, Green Ch=SGK1). (n=3). **(D)** Graphical representation of the proposed model.

Having shown that RANBP1 is endogenously correlated to both early and late Th17^+^ differentiation phase and that RANBP1 fluctuations are able of conditioning the fate of this differentiative process by ultimately modulating SGK1-dependent signaling, we wondered through which mechanism RANBP1 may affect the IL-23R-SGK1-FOXO1-RORγt signal transduction (17, 21, 22, 24). RANBP1, during interphase, controls nuclear import/export by activating the RANGAP1-dependent GTP-hydrolytic activity on RAN (26, 30, 31). Under IL-23R activation, SGK1 has been shown to regulate phosphorylation of FOXO1 by inducing its cytoplasmic re-localization, thereby allowing the activation of RORγt-dependent transcriptional function directed toward its target genes, IL-17A and IL-23R (23, 24). However, it is unclear how phosphorylation of FOXO1 justifies the SGK1 specificity in the regulation of differentiation process, since the SGK1-dependent FOXO1 phosphorylation-site is potentially common to other similar kinases (32). Previously, we have shown that SGK1 works as a potent activator of RANBP1 transcription (25), thus modulating mitotic stability (28) and nuclear transport of pre-miRNAs in interphase (27). To validate whether RANBP1-mediated mechanisms could underlie the specificity of SGK1 on the Th17^+^ differentiation process, we again transduced primary CD4^+^ with viral vectors inducing either RANBP1 over-expression or silencing. Cells were then used to study differential nuclear-cytoplasmic protein expression and immunofluorescence on transduced intact cells. When RANBP1 was over-expressed, we observed a depletion of the nuclear fraction of FOXO1 (0.3 ± 0.12 A.U.) with a concomitant increase in the cytoplasmic fraction (1.77 ± 0.023 A.U.) compared to control cells (**Figure 4A** left, **Supplementary Figures 3A, C**, **Supplementary Table 2**). The same evidence was obtained by immunofluorescence of intact cells (**Figure 4A** right) in which FOXO1 expression was totally extra-nuclear (**Supplementary Figure 4B** for imaging quantification). In contrast, when RANBP1 was silenced (**Figure 4B** left, **Supplementary Figures 3B, D**), nuclear FOXO1 fraction was strongly increased (1.67 ± 0.1 A.U.) with a concomitant decrease in the cytoplasmic proportion (0.78 ± 0.016 A.U.). This was evident both in differential proteins extraction through western blot analysis (**Figure 4B** left, **Supplementary Table 2**) and in localization study through immunofluorescence (**Figure 4B** right, **Supplementary Figure 4C** for imaging quantification). The same data observed in basal conditions were confirmed in primary Th17^+^ cell stimulated by 40mM NaCl (**Supplementary Figure 3**, **Supplementary Table 2**). We, therefore, wondered whether the FOXO1-relocalization attributed to RANBP1 was also consistent with SGK1-dependent FOXO1 regulation within the Th17^+^ differentiative signaling. In SGK1 over-expressing cells FOXO1 was mainly extranuclear, as expected (**Figure 4C** upper panel) (24). However, when SGK1 over-expression was associated with RANBP1 silencing, the SGK1-dependent cytoplasmic re-localization of FOXO1 was completely reversed (**Figure 4C** bottom panel, **Supplementary Figures 4D, E**).

## 4 Discussion

High Th17^+^ plasticity underlies the functional characteristics of this cluster of innate immunity lymphocytes (33). SGK1, both in the presence or absence of NaCl stimulation, is currently the leading candidate of IL-23R-dependent differentiative mechanism. Previous evidence has shown that SGK1 is a key regulator of Th17^+^/Treg differentiation through FOXO1 phosphorylation (8, 20, 33). It is still difficult to understand how the phosphorylation of a FOXO1 conserved residue recognized by several AGC family kinases could justify SGK1’s specificity (32). Our previous data have shown that SGK1 affects nuclear transport and mitotic stability by regulating the expression of RANBP1 (25). RANBP1 appears to be involved in several physiological processes (regulation of protein and miRNA transport, spindle checkpoint formation, and mitotic spindle nucleation) and in pathological dysregulation (e.g. tumors, neurocognitive deficits, or cellular alterations in the presence of viral and bacterial infections) (26). We hypothesize that RANBP1 represents the last missing element in the IL-23R-mediated Th17^+^ differentiative pathway by mediating the SGK1-dependent effects. Indeed, RANBP1 together with SGK1 are synchronously modulated in CD4^+^ lymphocytes during Th17^+^ differentiation. The role of SGK1 in Th17^+^ differentiation of lymphocytes is widely accepted (8, 16, 17, 24). Here, we demonstrate that the effects of SGK1 on Th17^+^ differentiation are mediated by RANBP1. We present evidence that RANBP1 is downstream in the SGK1-dependent Th17^+^ differentiation pathway and that, in this process, SGK1 requires the presence of RANBP1 (34). RANBP1 is essential for nucleus/cytoplasmic translocation and degradation of FOXO1, a direct antagonist of the RORγt-Th17^+^ differentiation program (35). Interestingly, this signaling pathway seems to be related to the “so called” pathological Th17^+^ response defined by the expression of high levels of IL-17 together with low levels of IL-10 (7). NaCl stimulation, in our experimental conditions, elevates Th17^+^ differentiation of naïve CD4^+^. Nevertheless, it also boosts FOXP3 levels, both transcriptionally and translationally, as observed from others (10). This generates a hybrid-situation of Th17^+^ RORγt/FOXP3 double-positive (36). However, as demonstrated, the influence of NaCl is significantly decreased when RANBP1/SGK1 is either overexpressed or silenced. Indeed, the dominance of the SGK1/RANBP1 pathway results in a shift towards a pathological phenotype of Th17^+^ switch, marked by high levels of RORγt and IL-17 and low levels of FOXP3 and IL-10. It seems that a dual regulatory level is established, where NaCl can enhance Th17^+^ differentiation and expression levels of SGK1 and RANBP1 up to a certain extent, but, in turn, higher levels of the proteins can lead to a NaCl-independent pathological differentiation. The identification of RANBP1 expression as a limiting step in Th17^+^ differentiation may contribute to a better understanding of the mechanisms underlying Th17^+^-mediated plasticity and to the identification of novel potential therapeutic targets for the treatment of diseases of immunity and inflammation (**Figure 4D**).

## Data availability statement

The raw data supporting the conclusions of this article will be made available by the authors, without undue reservation.

## Ethics statement

The studies involving human participants were reviewed and approved by ethics committee of the University ‘Magna Graecia’ of Catanzaro (r.s. Ethical Protocol register Id. 269, 24 Oct 2017). The patients/participants provided their written informed consent to participate in this study.

## Author contributions

CB conceptualization, Methodology, Formal Analysis, and Investigations. VD conceptualization, Methodology, Formal Analysis, and Investigations. LD conceptualization, Methodology, Formal Analysis, and Investigations. EC Methodology, Formal Analysis, and Investigations. RT Methodology, Formal Analysis, and Investigations. SA Methodology, Formal Analysis, and Investigations. VR Methodology. RI visualization. FT visualization. NP visualization and supervision. RA conceptualization, Methodology, Validation, Formal Analysis, Investigations, resources, data curation, writing-original draft preparation, writing-review-editing, visualization, and supervision. All authors contributed to the article and approved the submitted version.
