# Supplementary material for: RANBP1, a member of the nuclear-cytoplasmic trafficking-regulator complex, is the terminal-striking point of the SGK1-dependent Th17+ pathological differentiation
Source: Front Immunol. 2023 Jun 27;14:1213805. doi: 10.3389/fimmu.2023.1213805 (PMC10333757; doi:10.3389/fimmu.2023.1213805)
Supplement: Supplementary file 1 [file DataSheet_1.pdf]

frontiers

Supplementary Material

RANBP1, member of the nuclear-cytoplasmic trafficking-regulator complex, is the terminal-striking point of the SGK1-dependent Th17$^+$ pathological differentiation.

Brescia C [1,2 †], Dattilo V [3 †], D'Antona L [1,4 †], Chiarella E [3], Tallerico R [5], Audia S [1,2], Rocca V [1,4], Iuliano R [1,4], Trapasso F [3,4], Perrotti N [1,4] and Amato R [1,2,4, *]

[1] Department of Health Science, Medical School, University "Magna Graecia" of Catanzaro, Catanzaro, Italy

[2]Immuno-Genetics Lab, Department of Health Science, Medical School, University "Magna Graecia" of Catanzaro, Catanzaro, Italy

[3] Department Experimental and Clinical Medicine, Medical School, University "Magna Graecia" of Catanzaro, Catanzaro, Italy

[4] Medical Genetics Unit, University Hospital, Medical School, University "Magna Graecia" of Catanzaro, Catanzaro, Italy

[5] Microbiology and Virology Unit, "Pugliese-Ciaccio" hospital, Catanzaro, Italy

[†] These authors share first authorship

* Corresponding Author: rosario.amato@unicz.it (A.R.),

1 Supplementary Figures and Tables

1.1 Supplementary Figures

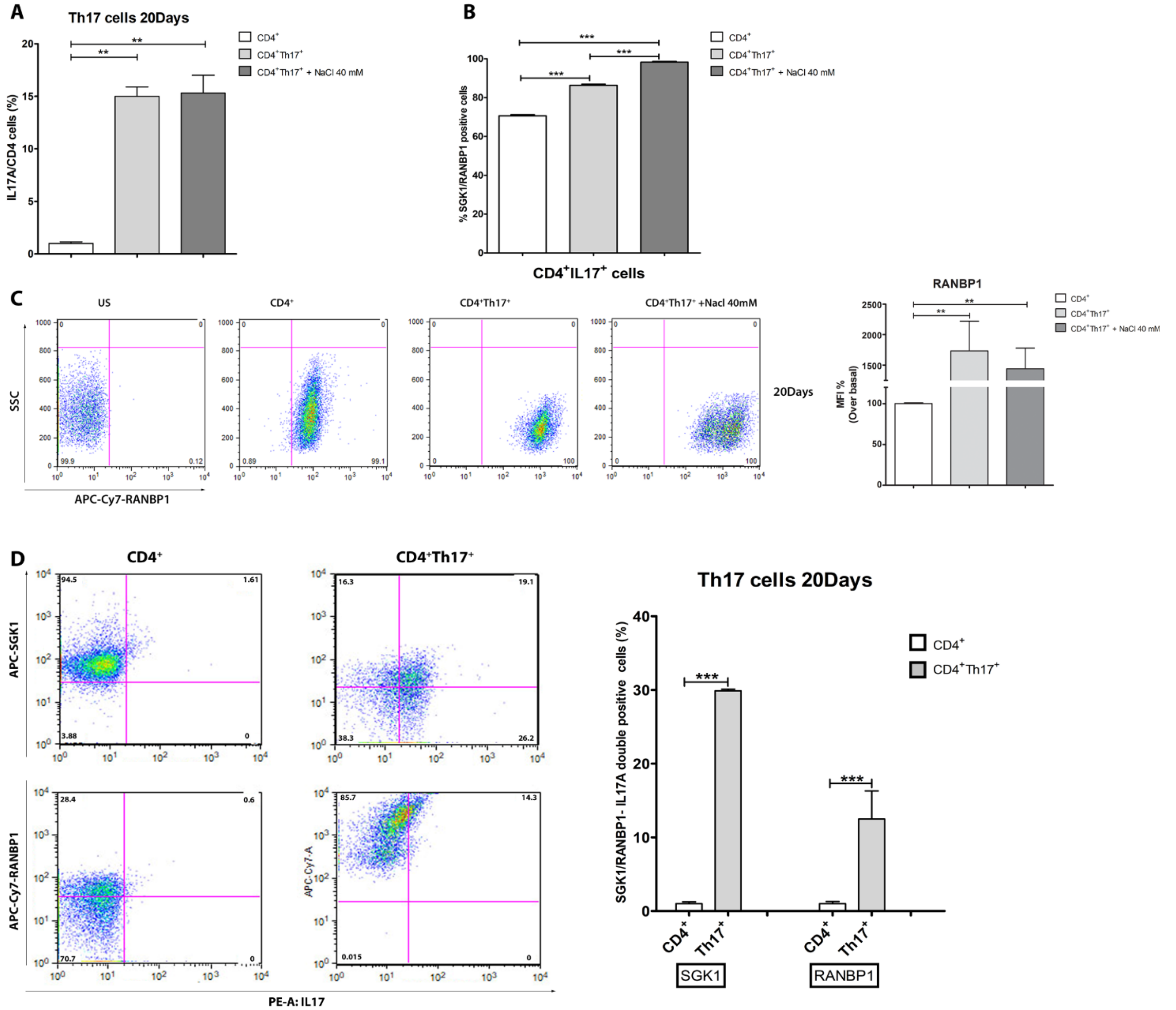

Supplementary Figure 1. Expression of SGK1, RANBP1 and IL-17A during Th17^{+} differentiation at 5 and 20 days. Related to Figure 1

A) FACS graphs and statistical analysis of IL-17A/CD4^{+} expression in CD4^{+} naïve (Th0) control cells, in basal and under 40mM NaCl stimulation CD4^{+}Th17^{+} cells, at 20 days of differentiation. (n=3)

B) FACS graphs and statistical analysis of SGK1/RANBP1 co-expression in CD4^{+} naïve (Th0) control cells, in basal and under 40mM NaCl stimulation CD4^{+}Th17^{+} cells, at 5 days of differentiation. (n=3)

C) Flow cytometric analysis of RANBP1 expression (versus physical parameter-SSC) in $CD4^+$ naïve (Th0) control cells, in basal and under 40mM NaCl stimulation $CD4^+Th17^+$ cells, at 20 days of differentiation. (n=3)

D) Flow cytometric analysis of RANBP1 and SGK1 in IL-17A expressing lymphocyte, in $CD4^+$ naïve (Th0) control cells and basal $CD4^+Th17^+$ cells, at 20 days of differentiation. (n=3)

Data are representative of at least three independent experiments (unless otherwise specified) and are shown as mean ± SD. *p <0.05 **p < 0.01 and *** p < 0.001 determined by one-way ANOVA followed by Bonferroni's post hoc test.

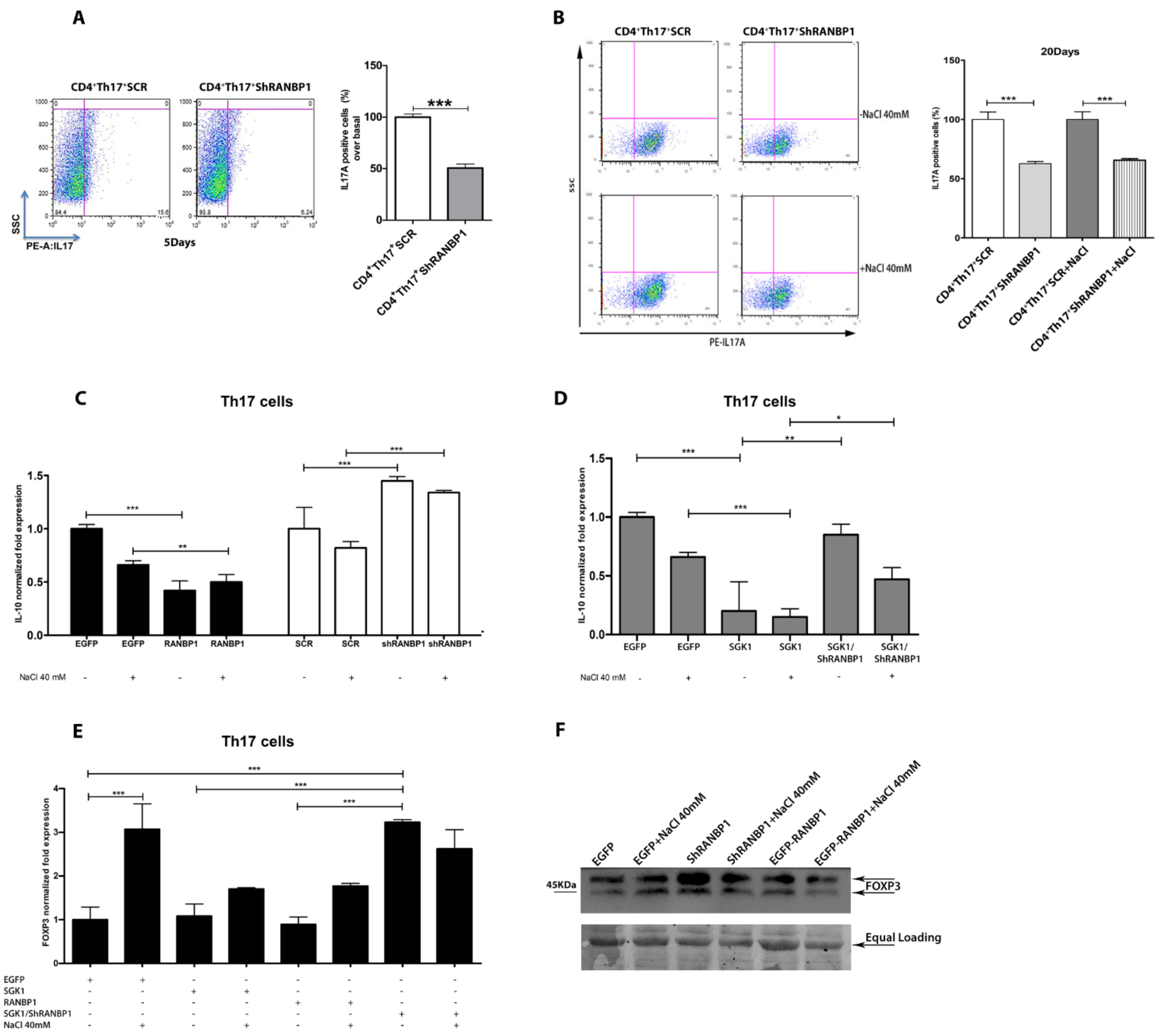

Supplementary Figure 2. Evaluation of IL-17A expression in primary Th17$^+$ cells silenced for RANBP1 at 5 and 20 days of differentiation, evaluation of IL-10 expression to determine the physiological/pathological phenotype of Th17$^+$ lymphocytes and qPCR and immunoblot analysis to assess the expression of FOXP3. Related to Figure 2 and 3

A-B) Flow cytometric analysis for IL-17A expression in RANBP1-silenced CD4$^+$Th17$^+$ cells compared to scrambled CD4$^+$Th17$^+$ control cells, in basal and stimulated cells, at 5 and 20 days of differentiation. (n=3)

C) qPCR analysis to assess the expression of IL-10 in control CD4$^+$Th17$^+$ cells compared to basal and NaCl-stimulated CD4$^+$Th17$^+$ cells, in presence of RANBP1 over-expression (left panel) or silencing by lentiviral-mediated approach (right panel). (n=3)

D) qPCR analysis to assess the expression of IL-10 in control $CD4^+Th17^+$ EGFP-cells compared to basal and NaCl-stimulated $CD4^+Th17^+$ cells, under SGK1 lentiviral over-expression, +/- concomitant RANBP1 lentiviral mediated Silencing, at 5 days of differentiation. (n=3)

E) qPCR and F) immunoblot analysis to assess the expression of FOXP3 (under either SGK1 or RANBP1 lentiviral over-expression, +/- concomitant RANBP1 silencing via lentiviral approach in basal and under 40mM NaCl stimulation CD4+Th17+ cells, at 5 days of differentiation (n=2; n=2).

Data are representative of at least three independent experiments (unless otherwise specified) and are shown as mean ± SD. *p <0.05 **p < 0.01 and *** p < 0.001 determined by one-way ANOVA followed by Bonferroni's post hoc test.

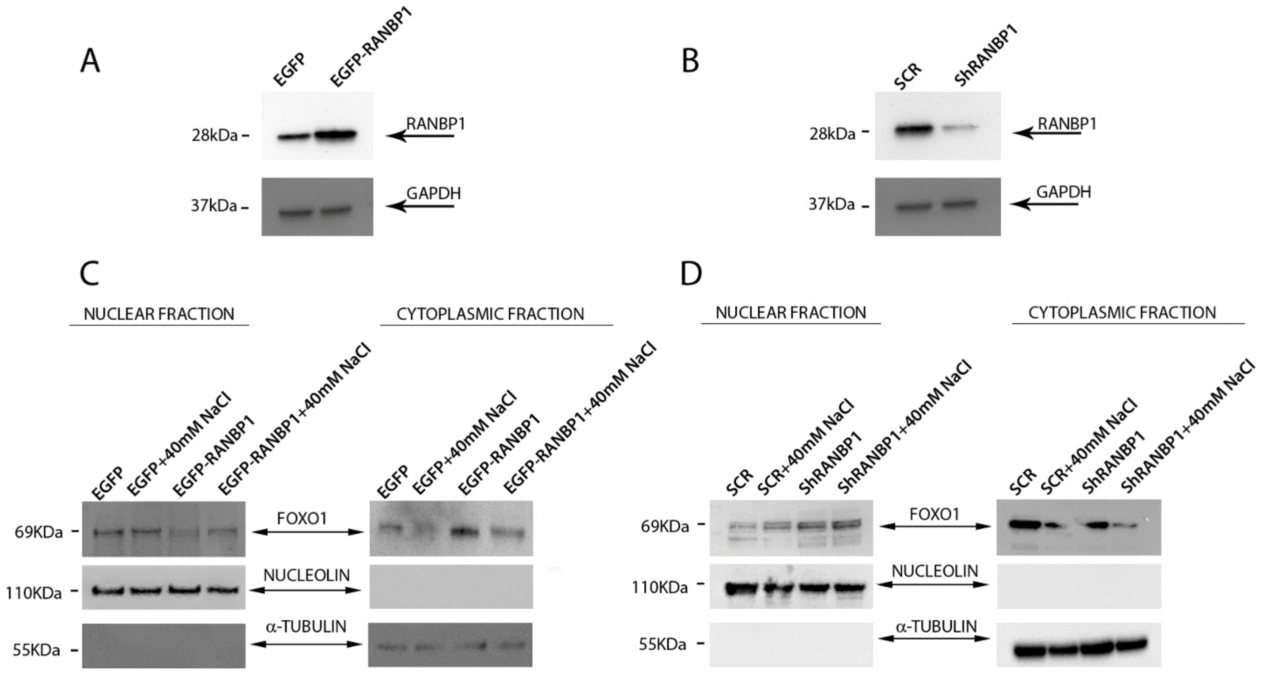

Supplementary Figure 3. Immunoblot analysis of FOXO1 expression in separate cytoplasmic and nuclear fractions in primary Th17$^+$ cells over-expressed or silenced for RANBP1. Related to Figure 4

A) Immunoblot analysis to assess RANBP1 over-expression in EGFP-RANBP1-CD4$^+$Th17$^+$ cells compared to control CD4$^+$Th17$^+$ (EGFP) cells. (n=2).

B) Immunoblot analysis to assess RANBP1 silencing in ShRANBP1-CD4$^+$Th17$^+$ cells compared to control CD4$^+$Th17$^+$ cells (SCR). (n=2).

C) Immunoblot analysis of FOXO1 expression in the separated cytoplasmic and nuclear fractions (un-cropped gel), under RANBP1 lentiviral over-expression in CD4$^+$ naïve (Th0) control cells and in basal CD4$^+$Th17$^+$ cells, at 5 days of differentiation. α-Tubulin and Nucleolin were used as cytoplasmic and nuclear loading controls, respectively. (n=2).

D) Immunoblot analysis of FOXO1 expression in the separated cytoplasmic and nuclear fractions (un-cropped gel), under RANBP1 lentiviral silencing in CD4$^+$ naïve (Th0) control cells and in basal CD4$^+$Th17$^+$ cells, at 5 days of differentiation. α-Tubulin and Nucleolin were used as cytoplasmic and nuclear loading controls, respectively.

Data are representative of at least three independent experiments (unless otherwise specified) and are shown as mean ± SD. $*p < 0.05$ $**p < 0.01$ and $***p < 0.001$ determined by one-way ANOVA followed by Bonferroni's post hoc test.

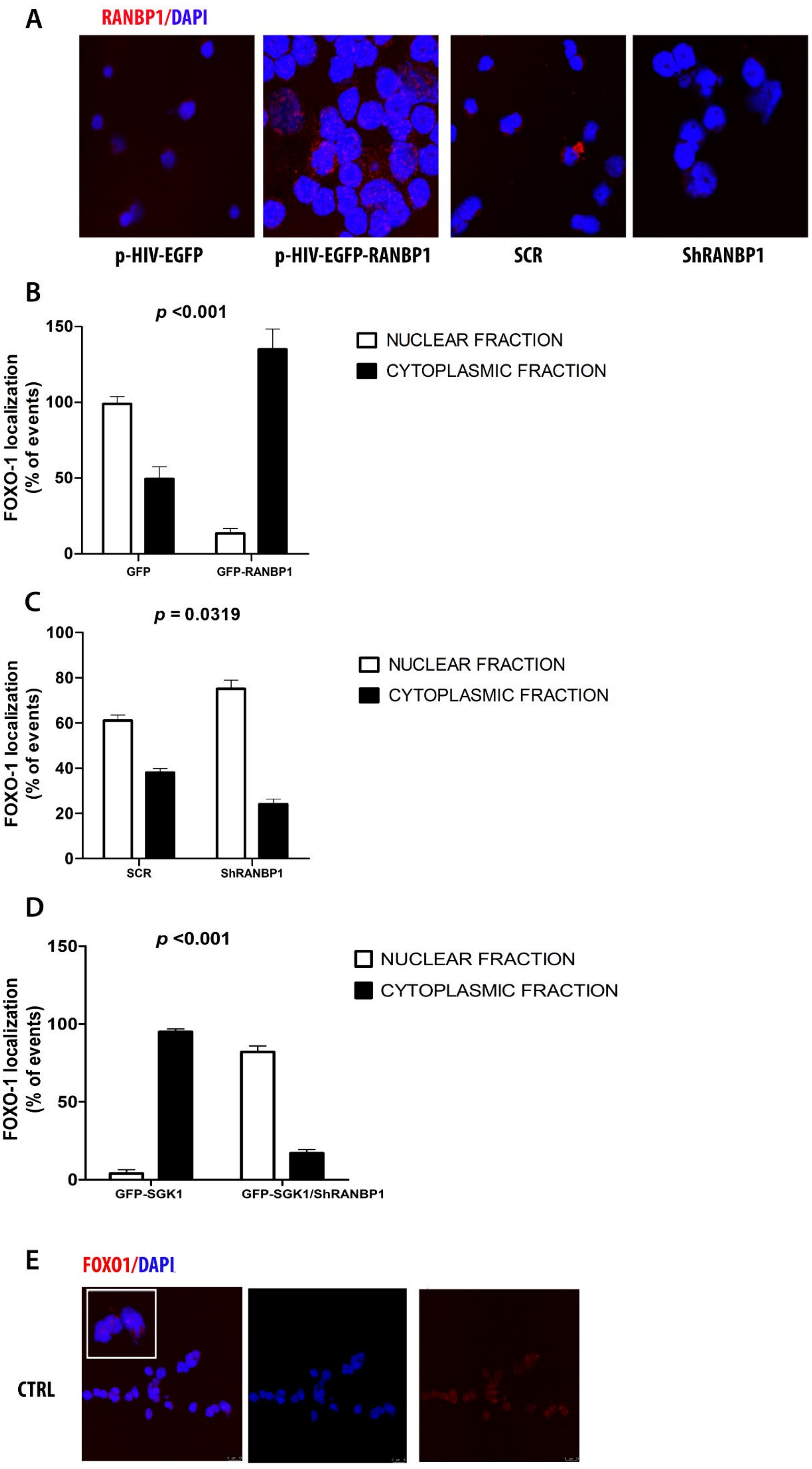
A
RANBP1/DAPI
p-HIV-EGFP
p-HIV-EGFP-RANBP1
SCR
ShRANBP1
B
p <0.001
FOXO-1 localization (% of events)
150
100
50
0
GFP
GFP-RANBP1
NUCLEAR FRACTION
CYTOPLASMIC FRACTION
C
p = 0.0319
FOXO-1 localization (% of events)
100
80
60
40
20
0
SCR
ShRANBP1
NUCLEAR FRACTION
CYTOPLASMIC FRACTION
D
p <0.001
FOXO-1 localization (% of events)
150
100
50
0
GFP-SGK1
GFP-SGK1/ShRANBP1
NUCLEAR FRACTION
CYTOPLASMIC FRACTION
E
FOXO1/DAPI
CTRL

Supplementary Figure 4. Immunofluorescence images and statistical graphs to assess the nuclear-cytoplasmic localisation of FOXO1 and RANBP1 in primary Th17$^+$ cells over-expressed/silenced for RANBP1 and overexpressed for SGK1 with concomitant RANBP1 lentiviral mediated Silencing. Related to Figure 4

A) Immunofluorescence analysis showing RANBP1 expression and localisation in either control EGFP-CD4$^+$Th17$^+$ cells and in EGFP-RANBP1-CD4$^+$Th17$^+$ cells or SCR-CD4$^+$Th17$^+$ cells and ShRANBP1- CD4$^+$Th17$^+$ cells (n=2).

B) Immunofluorescence statistical analysis for FOXO1 localisation in control EGFP-CD4$^+$Th17$^+$ cells compared to EGFP-RANBP1-CD4$^+$Th17$^+$ cells. (n=2).

C) Immunofluorescence statistical analysis for FOXO1 localisation in control SCR-CD4$^+$Th17$^+$ cells compared to ShRANBP1- CD4$^+$Th17$^+$ cells (n=2).

D) Immunofluorescence statistical analysis for FOXO1 localisation in EGFP-SGK1-CD4$^+$Th17$^+$ cells compared EGFP-SGK1-CD4$^+$Th17$^+$ cells with concomitant RANBP1 lentiviral mediated Silencing (n=2)

E) Immunofluorescence analysis to assess FOXO1 localisation in control CD4$^+$Th17$^+$ cells (n=2).

Data are representative of at least two independent experiments (unless otherwise specified) with 50 independent microscope fields counted for each experiment and are shown as mean ± SD. *p <0.05 **p < 0.01 and *** p < 0.001 determined by two-sided Chi-square (and Fisher 's exact) test.

1.2 Supplementary Table

Supplementary Table 1. Table summarizing all reagents and resources used in the whole experimental process.

REAGENT or RESOURCE	SOURCE	IDENTIFIER
**Antibodies**		
RANBP1	Santa Cruz Biotechonology	Sc-1160<br><br>Sc-374352
SGK1	Millipore Corporation, CA, USA	#07-315 EDM
p-SGK1	Cell Signaling Technology	#D36D11
IL-23R	Thermo Fisher Scientific	#PA5-113004
RORγ (D-4)	Santa Cruz Biotechonology	sc-365476
RORγt (AFKJS-9)	Thermo Fisher Scientific	#14-6988-82
Nucleolin (D4C70)	Cell Signaling technology	#14574
FOXO1	Thermo Fisher Scientific	#PA5-20972
p-FOXO1	Thermo Fisher Scientific	#PA5-17907
FOXP3	Cell Signaling technology	#5298
GAPDH	Santa Cruz Biotechnology	sc-47724

α Tubulin (DM1A):	Santa Cruz Biotechnology	sc-32293
Bovine anti-goat IgG-HRP	Santa Cruz Biotechnology	sc-2350-CM
Anti-mouse IgG HRP-linked	Cell Signaling Technology	#7076
Anti-rabbit IgG HRP-linked	Cell Signaling Technology	#7074
Goat anti-Rat IgG2a Secondary Antibody	Thermo Fisher Scientific	# PA1-84755
Alexa Fluor 594 AffiniPure Donkey Anti-Rabbit IgG (H+L)	Jackson Immunoresearch	711-585-152
Alexa Fluor 594 AffiniPure $F(ab')_2$ Fragment Donkey Anti-Goat IgG (H+L)	Jackson Immunoresearch	705-586-147
PE anti-IL17A (-clone CZ8-23G1)/IgG1k antibody	MiltenyiBiotec	130-120-410
Alexa Fluor 647 anti-SGK1(clone G-4) monoclonal antibody	Santa Cruz Biotechonology	SC-277360<br>AF647
RANBP1 Polyclonal Antibody, Cy5.5 Conjugated	Bioss Antibodies	bs-9322R-Cy5.5
FITC anti-human CD4 Antibody	Biolegend	Cat 300506
CD4 Antibody, anti-human,PE, REAfinity	Miltenyi Biotec	130-113-787

**Bacterial and virus strains**		
RANBP-1 shRNA (h) (200µl)	Santa Cruz Biotechonology	sc-41848-V
ShScr	Sigma-Aldrich	SHC002V
**Biological samples**		
FBS	Sigma-Aldrich	YOURSIAL-FBS-SA
Buffy Coat 50ml (blood from donors)	"Pugliese Ciaccio" Hospital Catanzaro. Italy	N/A
**Chemicals, peptides, and recombinant proteins**		
PBS1X	Gibco	10010-015
RPMI 1640	Life Technologies - Thermo Fisher Scientific	10-040-CV
Penicillin/Streptomycin	Sigma-Aldrich	P0781
Histopaque-1077	Sigma-Aldrich	S-10771
$CD4^+$ T Cell Isolation Kit human	Miltenyi Biotec	130-096-533
Lipofectamine 2000	Life Technologies - Thermo Fisher Scientific	11668-019
OPTI-MEM	Gibco	31985062
Polybrene	Sigma-Aldrich	107689-10G
Interleukin-6 human	MiltenyiBiotec	130-093-929
Interleukin-2 human	Sigma-Aldrich	I2644-10UG

TGF-β1	MiltenyiBiotec	130-095-066
DNAbeads Human T-Act CD3 / CD28	Life Technologies - Thermo Fisher Scientific	11131D
NaCl	Sigma-Aldrich	S3014-1KG
PMA	Sigma-Aldrich	Cat# P1585
Ionomycin	Sigma-Aldrich	Cat# I0634
BFA	Sigma-Aldrich	B7450
Tris-HCl pH 8 1M	Life Technologies - Thermo Fisher Scientific	15568025
IGEPAL CA-630	Sigma-Aldrich	I-3021
NaF (Sodium fluoride solution)	Sigma-Aldrich	201154-100G
DTT (Dithiothreitol)	Sigma-Aldrich	D9779-5G
Na_3VO_4 (Sodium orthovanadate)	Sigma-Aldrich	S6508-10G
PMSF (Phenylmethylsulfonyl fluoride)	Sigma-Aldrich	P7626-5G
Aprotinin from bovine lung	Sigma-Aldrich	A6279-10ML
HEPES	Sigma-Aldrich	H4034-100G
$MgCl_2$ (Magnesium chloride)	Sigma-Aldrich	M8266-100G
KCl (Potassium chloride)	Sigma-Aldrich	P9541-1KG
Glycerol	Sigma-Aldrich	G5516-1L
EDTA 0,5M pH 8	Gibco	15575-038

BSA (Bovine Serum Albumin)	Sigma-Aldrich	A-9647
TRITON X-100	Sigma-Aldrich	9002-93-1
Tween-20	Sigma-Aldrich	P7949-500ML
4-12% Nupage precast gel	Thermo Fisher Scientific	NW04120BOX
NuPAGE MES SDS Running Buffer (20X)	Thermo Fisher Scientific	B0002
NuPAGE Sample Reducing Agent (10X)	Thermo Fisher Scientific	B0009
NuPAGE LDS Sample Buffer (4X)	Thermo Fisher Scientific	NP0007
NuPAGE Antioxidant	Thermo Fisher Scientific	NP0005
Trans-Blot kit Turbo Transfer packs 0.2 µm PVDF, single application	BIORAD	#1704156
Blotting-Grade Blocker	BIORAD	#170-6404
ECL WESTERN BLOTTING DETECTION REAGENTS.	Cytiva	GEHRPN2209
4',6-diamidine-2-phenylindole (DAPI)	Thermo Fisher Scientific	D1306
Paraformaldehyde	Sigma-Aldrich	158127-100G
BD Cytofix/Cytoperm solution	BD Biosciences	554714
QIAzol Lysis Reagent	QIAGEN	1023537
**Critical commercial assays**		

RNA isolation RNeasy Mini Kit	Qiagen, Valencia, CA, USA	74104
High Capacity RNA-to-cDNA Kit	Applied Biosystems, Foster City, CA, USA	4387406
GoTaq (R) qPCR Master Mix kit	Promega	A6001
**Experimental models: Cell lines**		
HEK293T	ATCC	CRL-3216
**Experimental models: Organisms/strains**		
Primary human $CD4^{+}Th17^{+}$ cells from donors	"Pugliese Ciaccio" Hospital Catanzaro. Italy	N/A
Primary human $CD4^{+}$ cells from donors	"Pugliese Ciaccio" Hospital Catanzaro. Italy	N/A
**Oligonucleotides**		
hsa-IL-23R<br>fwd: 5'- TACTGGCAGCCTTGGAGTTCA -3'<br>rev: 5'- TAAGGTGCCCTGTAGAGATGGA- 3'	Life Technologies - Thermo Fisher Scientific	N/A
hsa-IL-17A<br>fwd:5'-TACAACCGATCCACCTCACCTT-3'<br>rev: 5'- ACTTTGCCTCCCAGATCACAGA- 3'	Life Technologies - Thermo Fisher Scientific	(Spagnuolo et al.)
hsa-RORC<br>fwd: 5'- GCAGCGCTCCAACATCTTCT-3'<br>rev:5'- ACGTACTGAATGGCCTCGGT-3'	Life Technologies - Thermo Fisher Scientific	N/A

hsa-SGK1<br><br>fwd: 5’- GGCACCCTCACTTACTCCAG- 3’<br><br>rev: 5’- GGCAATCTTCTGAATAAAGTCGTT- 3’	Life Technologies - Thermo Fisher Scientific	(Dattilo et al.)
hsa-IL-10<br><br>fwd: 5’- TGCCTTCAGCAGSGTGAAGA- 3’<br><br>rev: 5’- GGTCTTGGTTCTCSGCTTGG- 3’	PRIMM	N/A
hsa-RANBP1<br><br>fwd: 5’- ATGCGGGCAAAACTGTTCCGAT- 3’<br><br>rev: 5’- ATGGCCCCTTTCTCCTTGTGCT- 3’	Life Technologies - Thermo Fisher Scientific	(Dattilo et al.)
hsa-FOXP3<br><br>fwd: 5’- GACAGGCCACATTTCATGCAC- 3’<br><br>rev: 5’- TGGTGTGAGGCTGATCATGG- 3’	Eurofins Genomics	N/A
hsa-HPRT1<br><br>fwd: 5’- TGACACTGGCAAAACAATGCA- 3’<br><br>rev: 5’- GGTCCTTTTCACCAGCAAGCT- 3’	Life Technologies - Thermo Fisher Scientific	(Vandesompele et al.)
**Recombinant DNA**		
pHIV-EGFP	Addgene	#21373
**Software and algorithms**		
FlowJo software version 9.3.1	BD Biosciences	
Prism	GraphPad	
Las X	Leica	
Uvitec Alliance Software	Uvitec Cambridge	

**Other**		
CFX96 Touch Real-Time OCR Detection System	Biorad	
NanoDrop 2000 / 2000c Spectrophotometer	Thermo Scientific	
TCS SP8 confocal microscope	Leica	
FACS CANTO II	BD Biosciences	
Uvitec Cambridge (Alliace mini 2M)	Uvitec Cambridge	

Supplementary Table 2. *Statistical densitometric analysis of immunoblottings*

Fig.2B

ID	$Th17^{+}$ *Mean* OVER $CD4^{+}$	SD	$Th17^{+}$ 40mM NaCl *Mean* OVER $CD4^{+}$	SD2
p-SGK1	1.64	0.05	1.94	0.13
SGK1	1.90	0.01	1.94	0.03
RANBP1	1.96	0.11	2.13	0.1
p-FOXO1	1.93	0.31	1.99	0.2
FOXO1	1.90	0.36	1.92	0.014
RORγ	1.91	0.12	1.94	0.12
GAPDH	1.01	0.0021	1.0	0.013

Fig.3B

ID	EGFP-RANBP1 *Mean* OVER EGFP	SD	EGFP-RANBP1 40mM NaCl *Mean* OVER EGFP 40mM NaCl	SD2	EGFP-RANBP1 *Mean* OVER EGFP-RANBP1 40mM NaCl	SD3
RANBP1	2.62	0.045	2.01	0.02	1.001	0.0071
IL-23R	1.97	0.07	1.5	0.13	0.99	0.15
RORγ	1.57	0.01	1.33	0.014	1.0	0.21
GAPDH	1.03	0.002	0.98	0.0035	1.0	0.001

Fig.3D

ID	Sh-RANBP1 *Mean* OVER SCR	SD	Sh-RANBP1 40mM NaCl *Mean* OVER SCR 40mM NaCl	SD2	Sh-RANBP1 *Mean* OVER Sh-RANBP1 40mM NaCl	SD3
RANBP1	0.52	0.07	0.44	0.037	1.04	0.14
IL-23R	0.40	0.032	0.55	0.2	0.99	0.11
RORγ	0.31	0.011	0.5	0.13	1.2	0.2
GAPDH	0.94	0.2	0.95	0.07	1.0	0.035

Fig.3F

ID	EGFP-SGK1 *Mean* OVER EGFP	SD	EGFP-SGK1 40mM NaCl *Mean* OVER EGFP 40mM NaCl	SD2	EGFP-SGK1/Sh-RANBP1 *Mean* OVER EGFP-SGK1	SD3	EGFP-SGK1/Sh-RANBP1 40mM NaCl *Mean* OVER EGFP-SGK1 40mM NaCl	SD4
RANBP1	1.0	0.11	1.45	0.23	0.37	0.045	0.2	0.07
SGK1	1.8	0.017	2.35	0.14	1.0	0.3	1.4	0.21
IL-23R	1.6	0.2	1.2	0.1	1.18	0.2	0.8	0.2
RORγ	1.83	0.12	2.2	0.11	1.2	0.15	0.7	0.31
GAPDH	1.07	0.13	1.13	0.11	0.9	0.2	0.88	0.23

Fig.4A and Suppl. Fig.3C

ID	EGFP-RANBP1 *Mean* OVER EGFP nuclear	SD	EGFP-RANBP1 40mM NaCl *Mean* OVER EGFP nuclear 40mM NaCl	SD2	EGFP-RANBP1 *Mean* OVER EGFP cytoplasmic	SD3	EGFP-RANBP1 40mM NaCl *Mean* OVER EGFP 40mM NaCl	SD4
FOXO1	0.33	0.12	0.78	0.014	1.77	0.023	1.3	0.031
α-Tubulin	-	-	-	-	1.03	0.12	1.1	0.13
Nucleolin	1.2	0.11	1.0	0.09	-	-	-	-

Fig.4B and Suppl. Fig.3D

ID	Sh-RANBP1 *Mean* OVER SCR nuclear	SD	Sh-RANBP1 40mM NaCl *Mean* OVER SCR nuclear 40mM NaCl	SD2	Sh-RANBP1 *Mean* OVER SCR cytoplasmic	SD3	Sh-RANBP1 40mM NaCl *Mean* OVER SCR 40mM NaCl cytoplasmic	SD4
FOXO1	1.67	0.1	1.3	0.019	0.78	0.016	0.65	0.13
α-Tubulin	-	-	-	-	1.0	0.2	0.8	0.124
Nucleolin	1.1	0.13	0.91	0.1	-	-	-	-
